# Biological Impact of Phenolic Compounds from Coffee on Colorectal Cancer

**DOI:** 10.3390/ph14080761

**Published:** 2021-08-03

**Authors:** Hernán Villota, Manuel Moreno-Ceballos, Gloria A. Santa-González, Diego Uribe, Isabel Cristina Henao Castañeda, Lina María Preciado, Johanna Pedroza-Díaz

**Affiliations:** 1 Biomedical Innovation and Research Group, Instituto Tecnologico Metropolitano, Faculty of Applied and Exact Sciences, Medellin 050012, Colombia; hernanvillota137600@correo.itm.edu.co (H.V.); manuelmoreno259164@correo.itm.edu.co (M.M.-C.); gloriasanta@itm.edu.co (G.A.S.-G.); diegouribe@itm.edu.co (D.U.); 2 Productos Naturales Marinos, Facultad de Ciencias Farmacéuticas y Alimentarias, Universidad de Antioquia UdeA, Medellín 050010, Colombia; isabel.henao@udea.edu.co; 3 Toxinología y Alternativas Terapéuticas y Alimentarias, Facultad de Ciencias Farmacéuticas y Alimentarias, Universidad de Antioquia UdeA, Medellín 050010, Colombia; maria.preciado@udea.edu.co

**Keywords:** natural compounds, phenolic compounds, cell biology, pharmaceutical science, coffee

## Abstract

Colorectal cancer is one of the leading death-related diseases worldwide, usually induced by a multifactorial and complex process, including genetic and epigenetic abnormalities and the impact of diet and lifestyle. In the present study, we evaluated the biological impact of two of the main coffee polyphenols, chlorogenic acid (CGA) and caffeic acid (CA), as well as two polyphenol-rich coffee extracts (green coffee extract and toasted coffee Extract) against SW480 and SW620 colorectal cancer cells. First, the total phenolic content and the antioxidant capability of the extracts were determined. Then, cytotoxicity was evaluated by MTT and SBR. Finally, a wound healing assay was performed to determine the impact on the cell migration process. The results showed a cytotoxic effect of all treatments in a time and dose-dependent manner, which decreased the viability in both cell lines at 24 h and 48 h; likewise, the migration capability of cells decreased with low doses of treatments. These results suggest the potential of coffee to modulate biological mechanisms involved in colorectal cancer development; however, more studies are required to understand the mechanistic insights of these observations.

## 1. Introduction

Colorectal cancer (CRC) is one of the most common and deadly diseases globally, with 1.9 million new cases and approximately 935,000 deaths in 2020 [1]. Incidence rates of CRC fluctuate worldwide, with the highest incidences found mainly in developed countries such as New Zealand, Australia, and the United States. On the other hand, countries with lower CRC incidences are found in Africa and Southwest Asia. This variation in incidence could be explained by differences in eating and cultural habits between regions [2].

Among the main risk factors for developing CRC are hereditary predisposition and environmental factors such as diet, exercise, smoking, alcohol consumption, and metabolic disorders such as obesity and type 2 diabetes [3]. In addition, the stage at which cancer disease is detected determines the prognosis, survival, and patient treatment. Unfortunately, most cases are diagnosed in advanced stages of the disease, thus resorting to systemic therapies like chemotherapeutic regimens, with a high probability of disease recurrence and adverse side effects that could decrease the patient’s life quality and adherence to treatment. [4,5,6]. In addition, tumor invasion and metastasis in the middle and late stages of the disease are recognized as the leading causes of treatment failure and poor therapeutic efficacy [7,8].

In this context, it is mandatory to carry out studies to develop new strategies for controlling and treating CRC. In this regard, it is known that despite the multifactorial etiology of this disease, consumption of red and processed meats increases the risk of developing CRC [9,10]. In contrast, the consumption of fruits, vegetables, and fiber have been proposed to decreases the risk of disease onset and progression [11,12]

Polyphenols are the most abundant antioxidants in plant-origin foods and beverages and have aroused interest in recent years as potential anticancer compounds. Specifically, it has been described that dietary polyphenols can modulate different processes in cancer cells, acting as blocking agents in the initial steps of tumor development, suppressors of disease progression, or both [13,14]. Different in vitro studies carried out on cells models of breast, gastric, liver, and colon cancer, among others, have shown the potential of different classes of polyphenols to modulate signaling pathways related to tumor development, invasion, and metastasis, such as Wnt/β-catenin, Hedgehog and Notch, and AKT/GSK3β [15,16]. This is interesting, considering that different epidemiological and experimental studies have linked coffee consumption with a reduced risk of suffering some types of cancer, including CRC. The effects could be related to its high polyphenolic content, which includes caffeic acid (CA; 3,4-dihydroxycinnamic acid) and chlorogenic acid (CGA; 3-*O*-caffeoylquinic acid) [17,18,19]. However, the molecular mechanism associated with this therapeutic effect has been poorly studied in CRC models.

**Figure 1:**
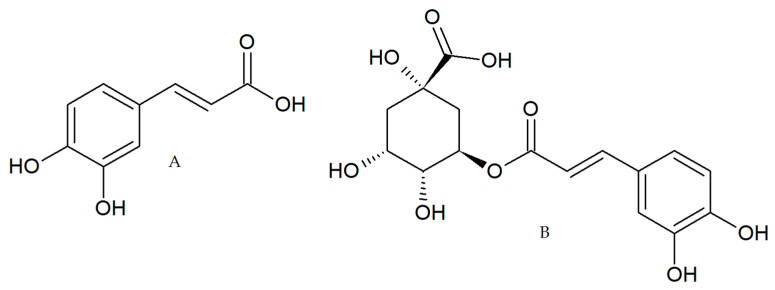
Caffeic acid, CA (**A**) chlorogenic acid, CGA (**B**).

In addition to its antioxidant activity, CA and CGA (Figure 1) showed anti-inflammatory effects in different models and the impairment of chemical carcinogenesis induction in animal models [20,21,22] These studies suggest the chemopreventive and anticancer properties of these polyphenols present in coffee; however, few studies have been performed in CRC models to elucidate the molecular mechanism involved. For these reasons, in the present study, we evaluated in tumor-derived CRC cell lines, the biological activity of two coffee extracts (green and toasted) from Coffee Arabica collected in the Andean mountains of Antioquia-Colombia and two polyphenols (CA and CGA) present in coffee.

## 2. Results

### 2.1. Antioxidant Activity and Total Phenolic Content of Colombian Coffee Extracts

**Figure 2:**
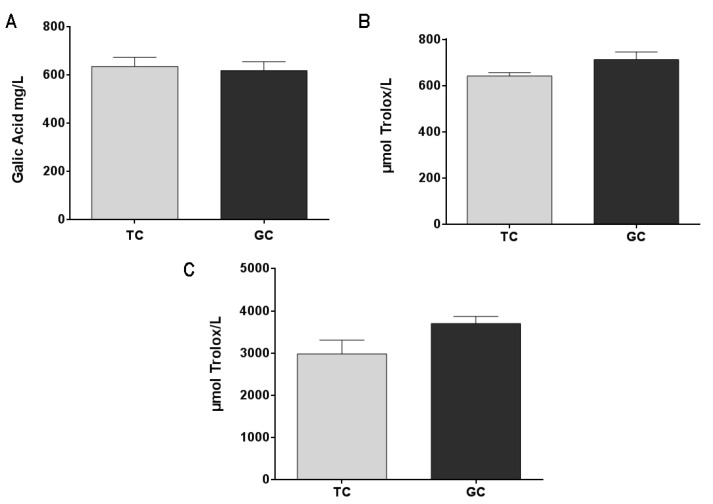
Total phenolic content (**A**) in green coffee extract (GC) and toasted coffee extract (TC) (results are expressed as gallic acid mg/L). Antioxidant capacity was measured by the DPPH Radical Scavenging capacity method (**B**) and by the FRAP method (**C**). Results are expressed as μmol Trolox equivalent/L.

Green coffee extract (GC) and toasted coffee extract (TC) are rich in polyphenols and present an important antioxidant capacity with no significant difference between them (Figure 2B,C). These results are correlated with the total phenolic content (Figure 2A) that also showed no significant differences between GC and TC extracts.

### 2.2. Cytotoxicity Assessment

**Figure 3:**
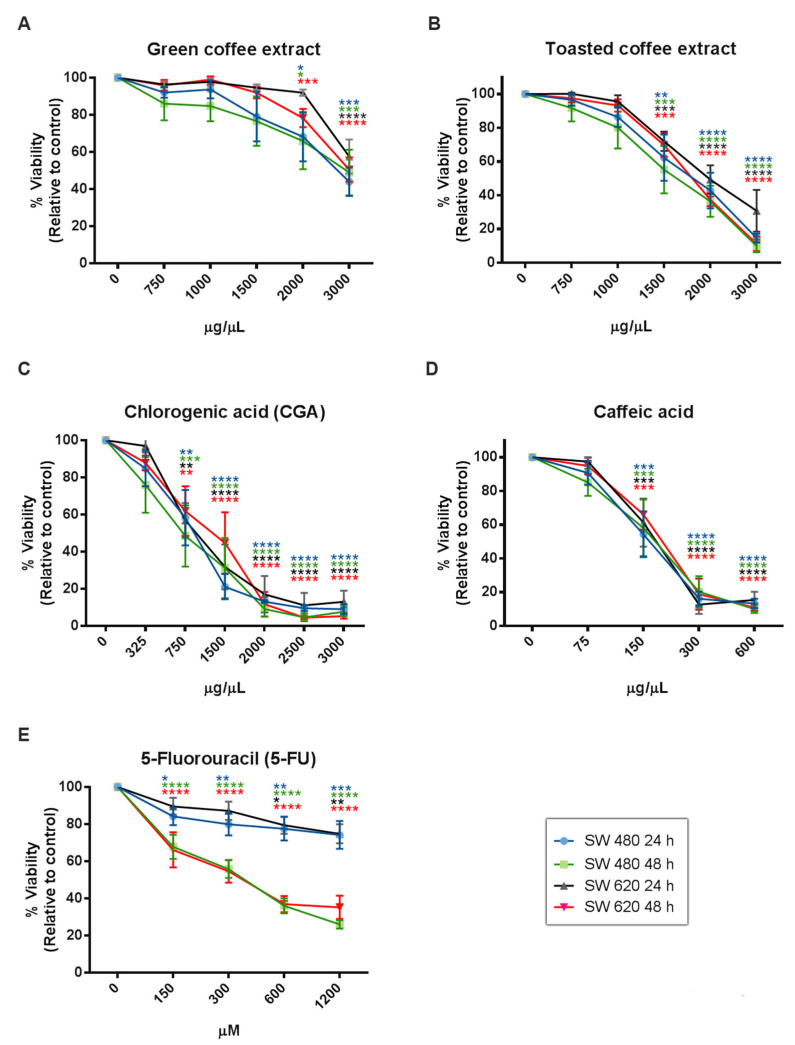
Cytotoxicity activity measured by MTT in the colorectal cancer cell lines SW480 and SW 620. GC (**A**), TC (**B**), CGA (**C**), CA (**D**), and 5-FU (**E**). Values are expressed as mean ± SEM of at least three independent experiments. Two-way ANOVA, difference to non-treated cells, * *p* ≤ 0.05, ** *p* ≤ 0.01, *** *p* ≤ 0.001, **** *p* ≤ 0.00001.

The cytotoxic effect of coffee extracts and the polyphenolic compounds evaluated by MTT assay (Figure 3) showed a statistically significant dose and time-dependent inhibition of cell viability from 2000 µg/mL for GC, 1500 µg/mL for TC, 750 µg/mL for CGA, and 150 µg/mL for CA. Likewise, IC_50_ values were calculated at 24 h and 48h of treatment (Table 1), and the results showed that SW620 cells are more tolerant to treatments than SW480 cells. Finally, 5-Fluorouracil (5-FU), a clinically used compound, showed higher IC_50_ values than CGA and CA, and lower IC_50_ values than GC and TC.

**Figure 4:**
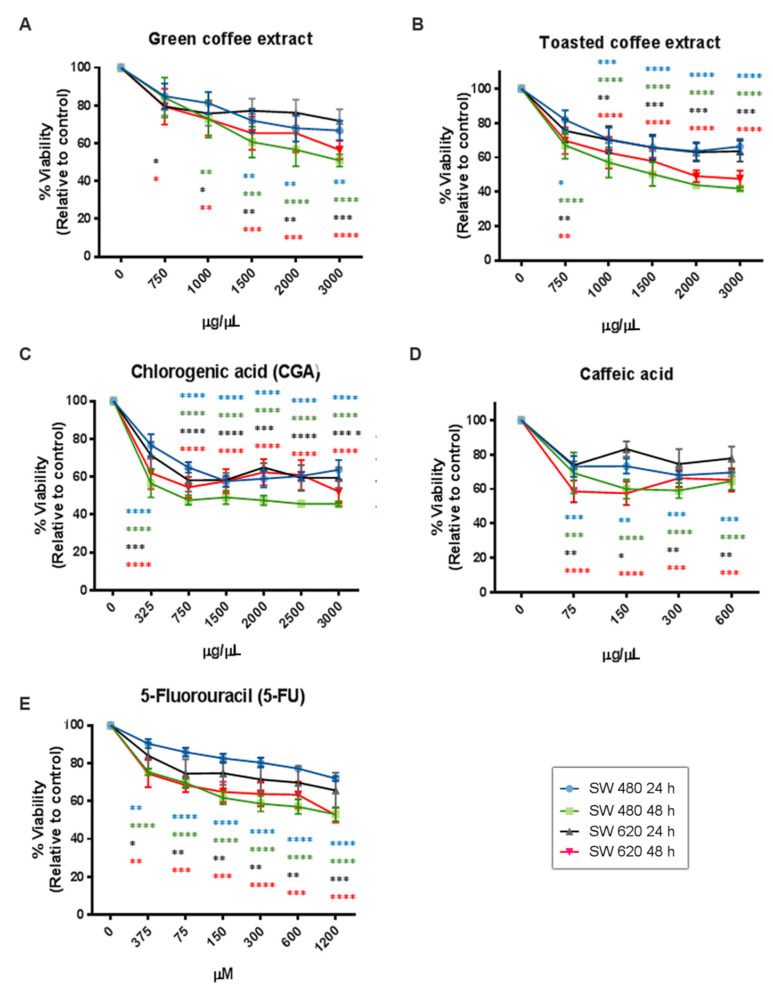
Cytotoxicity activity measured by SRB in the colorectal cancer cell lines SW480 and SW620. GC (**A**), TC (**B**), CGA (**C**), CA (**D**), and 5-Fu (**E**). Values are expressed as mean ± SEM of at least three independent experiments. Two-way ANOVA, difference to non-treated cells, * *p* ≤ 0.05, ** *p* ≤ 0.01, *** *p* ≤ 0.001, **** *p* ≤ 0.00001.

Several studies have reported that the antioxidant activity of phytochemicals could interfere with the MTT assay because this method requires the reduction of MTT to formazan by a mitochondrial enzyme [23,24]. For these reasons, we also used the Sulforhodamine B method (SRB) to avoid misinterpreted data. The SRB mechanism relates to this dye’s capacity to bind basic amino acid residues on proteins and determine its abundance in cells. SRB results (Figure 4) showed a dose/time-dependent cytotoxic effect and similar behavior to those obtained by the MTT method. However, some differences in the concentration-effect relationship were observed. We found IC_50_ values very close to MTT in SW480 cells, although IC_50_ values for the CGA and CA treatments increased (Table 1). In SW620 cells, the IC_50_ value of GC decreased while for CGA and CA increased. Essentially, the SRB assay seems to be more sensitive than the MTT assay because it presents a higher reproducibility [25]. However, it is important to consider that the MTT assay requires cellular metabolic activity to convert the tetrazolium (colorless) to formazan dye (purple-colored); therefore, it detects only viable cells.

In contrast, the SRB method does not distinguish between viable and dead cells, and it could show IC_50_ values slightly higher [26]. Likewise, the amount of metabolized MTT is not linear with cell number, while the SRB results have better linearity with cell density [27]. Additionally, it is important to note that both quantitation assays are widely used for cytotoxic screening evaluation of bioactive compounds and could be used as complementary techniques.

### 2.3. Cell Migration

Different cell concentrations were seeded in a 24-well plate, using 500 µL of medium with/without serum supplementation to determine the cell density required for establishing a monolayer at 90% of confluence after 48 h of cell seeding. Results showed that the cell number required to obtain the desired cell density was 8 × 10^5^ cells/mL in culture medium without SBF supplementation.

**Figure 5:**
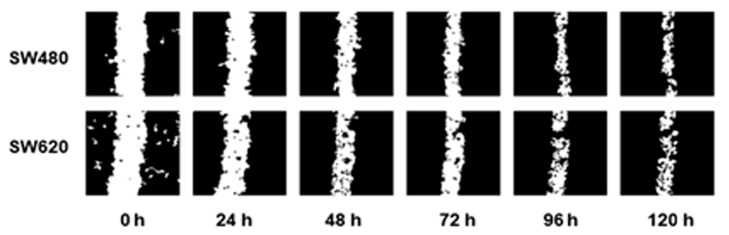
Migration pattern of the colorectal cancer cell lines SW480 and SW620 without treatments and without SBF supplementation. Images were captured in intervals of 24 h during 120 h.

Cell migration assays were performed, creating a scratch in the cell monolayer using a 10 µL pipette tip. The images were captured every 24 h until partial closure of the wound to determine the optimal time-duration of the assay (Figure 5).

Subtoxic concentrations of coffee extracts were used for wound healing treatments: 750 µg/mL (GC) and 500 µg/mL (TC) for SW480 cells, 1000 µg/mL (GC), and 750 µg/mL (TC) for SW620 cells. The concentrations used were the same for pure compounds in both cell lines: 187 µg/mL, 100 µg/mL, and 150 µg/mL for CGA, CA, and 5-FU, respectively.

**Figure 6:**
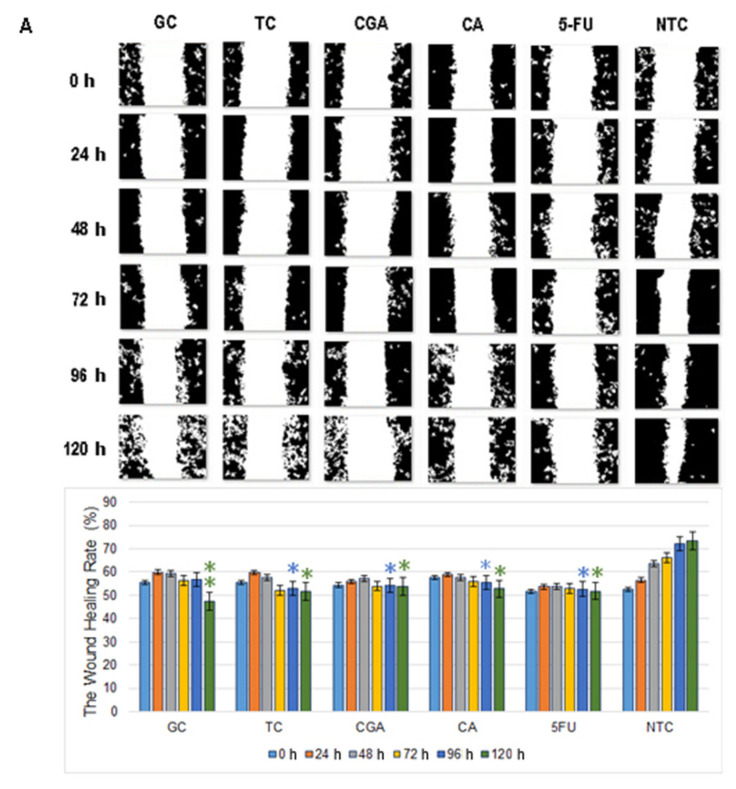

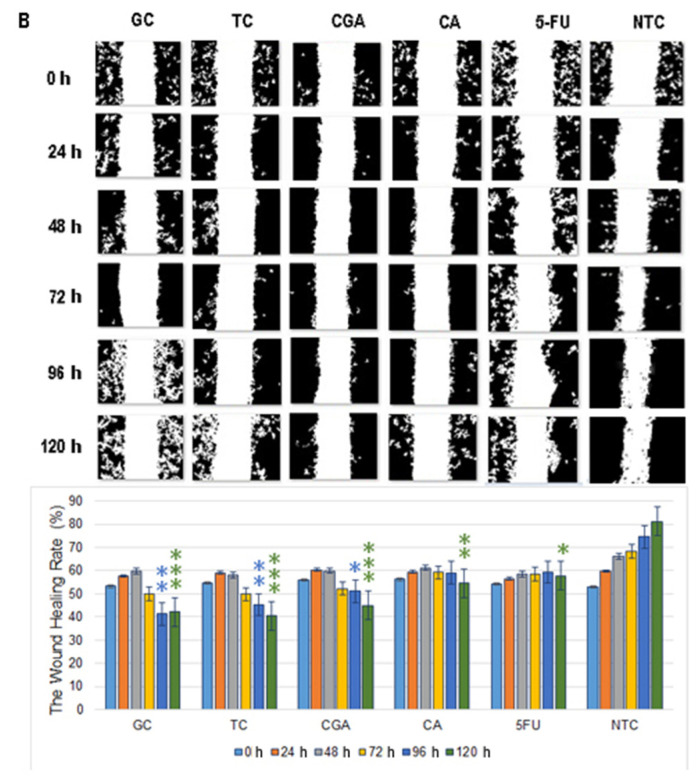
Migration patterns of SW480 (**A**) and SW620 (**B**) cell lines were evaluated by wound healing assay. Cell migration was observed in an inverted microscope (10 × magnification) at intervals of 24 h during 120 h. The quantitative analysis of cell migration in each group: from left to right 1, (GC), 2 (TC), 3 (CGA), 4 (CA), 5 (5-FU) and 6 (NTC) non-treated cells. Values are expressed as mean ± SEM of three independent experiments. Two-way ANOVA, the difference to non-treated cells, * *p* ≤ 0.05, ** *p* ≤ 0.01, *** *p* ≤ 0.001.

Wound healing assay revealed that the 120 h migration distance rates of both cell lines in all the treatments were significantly shorter than the NTC-control group. In SW480 cells, CGA and 5-FU treatments showed a non-migration pattern, while CA, TC, and GC treatments showed a small increment in the open wound area over time (Figure 6A).

In SW620 cells, CGA, TC, and GC treatments showed a more intensive increment in the open wound area. For CA and 5-FU treatments, a slow and minimums migration pattern was observed (Figure 6B). In both cell lines, the NTC-control group shows a wound healing rate of around ≈80%, while all the treatments could not get over ≈55%.

## 3. Discussion

CRC is the third most common cancer diagnosed and the second cancer-related death in the world. Furthermore, it is expected an increment by ≈60%, with approximately 3 million new cases and 1.5 million cancer deaths by 2040 [1]. Metastasis is the main cause of CRC-related dead. Therefore, it constitutes a serious concern, considering that about 22% of CRC are metastatic at the initial diagnosis, and about 70% of patients will develop metastatic relapse. Additionally, it has been observed that the 5-year survival rate for stage IV or metastatic colorectal cancer is only 12%, compared to the 71% and 90% for regional and localized CRC in the United States [8].

For these reasons, it is important to develop new strategies for the control and treatment of CRC. In this regard, substantial evidence has confirmed that different polyphenols and other natural compounds offer protection against different human diseases, such as metabolic diseases and various cancer types [14,17], including colorectal cancer.

Several studies have confirmed that CGA and CA are polyphenols included in green and toasted coffee. CGA is the main polyphenol in green coffee beans with a proportionaround 35.11%, with a similar concentration in robusta and arabica varieties (21–45 g/kg) [28,29]. On the other hand, CA presents a low concentration compared to CGA in green coffee beans, which is incremented during the roasted process due to the decomposition of caffeoylquinic acids [30]. Additionally, it is important to note that a sample of Colombian coffee had a mean of 51.240 mg/g of caffeoylquinic acids [31].

This study reports the cytotoxicity activity and the inhibition of the migration process of two rich polyphenolic coffee extracts and two polyphenolic compounds, known as CGA and CA, on SW480 and SW620 colorectal cancer cell lines. Treatments showed dose and time-dependent cytotoxic effects on both cell lines; as we expected, the SW620 cells were less sensitive to treatments, which could be related to metastatic characteristics of these cells compared with SW480, considering that SW480 cell line cells were established from an In situ colorectal carcinoma.

In other cancer models, such as MDA-MB-231 breast cancer cells, CA showed cytotoxic activity by inducing ≈66.4% reduction of cell viability at doses of 100 µM for 24 h [32]. However, few studies evaluate the effect of CA in colorectal cancer models, but there are some reports that asses the activity of its metabolites. For example, caffeic acid phenethyl ester (CAPE) presents IC_50_ values of 108 µM at 72 h on RKO colorectal carcinoma cells [33]. In addition, CA, ethyl caffeic acid (EC), and decyl caffeic acid (DC) showed IC_50_ values of 116 μM, 90 μM and 14 μM on HCT-116 cells, and 101 μM, 74 μM, and 10 μM on HT-29 cells [34]. Likewise, some studies report IC_50_ values for CGA of 758 µM at 24 h on Caco-2 cells [35] and 1000 µM at 72 h on HTC-116 and HT-29 cells [36].

In this context, when we compare our MTT results with previous reports on RKO, HCT-116, HT-29, and Caco-2 cells, CA and CGA showed less cytotoxic effects on SW480 and SW620 cell lines, when we did the conversion of our IC_50_ to µM equivalent units (CA 965 µM and CGA 1000 µM for SW480 cells; CA 1920 µM and CGA 2347 µM for SW620 cells) (Figure 3). Likewise, higher concentrations of 5- FU were required for IC_50_ in our cells models: 18 mM at 24 h and 2 mM at 48 h on SW480 cells and 22 mM at 24 h, and 7 mM at 48 h on SW620 cells (Table 1).

On the other hand, some studies reported the effects of polyphenols on the migratory properties of colorectal cancer cells. For example, resveratrol can inhibit the invasion and migration process by reversing EMT in SW480 and SW620 cells when treated for 48 h with 15 µM of resveratrol [16]. Other studies report CA activity on inhibiting the migration of oral carcinoma cells (SCC-25) at 50 µM for 48 h [37]. Likewise, dose-dependent inhibition of MDA-MB-231 breast cancer cells’ migration capabilities was observed with 50 µM and 100 µM of CA (wound area at 24 h was 9% and 6%, respectively) [32]. For CGA, it was observed the inhibition of liver cancer HepG2 cells migration at 48 h with 1000 µM [38], which is higher than the effect of the same compound on A549 lung cancer cells [39,40]. Likewise, in endothelial-derived cell models, e.g., HUVEC cells, it has been observed that cell motility induction under hypoxic conditions could be reversed by treatments with CGA at 10 µM for 24 h [41].

Our study shows that the migratory capabilities of SW480 and SW620 without treatments and SBF supplementation were similar, with a constant velocity of wound healing and a partial closure of wound at 120 h (Figure 5). Likewise, it was observed a decrease in the migration process with all treatments. Still, higher concentrations of CA and CGA (505 µM and 606 µM, respectively) were used when compared with previous reports in other cancer models (Figure 6). In GC and TC, a mild cytotoxic effect was observed as the exposure time to the extracts increased, causing the augmentation of the open-wound area.

## 4. Materials and Methods

### 4.1. Chemicals and Reagents

Chlorogenic acid ≥ 95% (tritation) Ref C3878-1G, Caffeic acid ≥ 98% (HPLC) Ref C0625-25G, 5-Fluorouracil ≥ 99% (HPLC) Ref F6627-5G, 3-(4,5-dimethylthiazol-2-yl)-2,5-diphenyltetrazolium bromide (MTT) were purchased from Sigma-Aldrich. Isopropyl alcohol from Merck; Acidified isopropyl alcohol, PBS, fetal bovine serum (FBS), Dulbecco’s modified eagle’s medium (DMEM), penicillin and streptomycin from GIBCO.

### 4.2. Coffee Extracts and Compounds

Two extracts of Coffee Arabica rich in polyphenols were prepared from green coffee beans and toasted coffee from Natucafé, a coffee farm located in Andes-Colombia. Coffee samples were powdered, and solid-liquid extractions were carried out using isopropanol:water 60:40 [39], over 48 h with three intervals of 30 min of sonication at room temperature and a sample-solvent ratio of 1:3. After the extractions, the solvent was evaporated in a rotary evaporator at 40 °C and then lyophilized and stored at −20 °C.

For treatments, 10 mL of ultrapure water were added to 200 mg of both lyophilized extracts, then aliquots of 20 mg/mL were used and stored at −20 °C. CGA, CA, and 5-FU were dissolved in the medium before treating the cells.

### 4.3. Coffee Extract Antioxidant Activity and Total Phenolic Content

DPPH radical scavenging capacity: 5 µL of 2,2-diphenyl-1-picrylhydrazyl (DPPH) methanolic solution at 10 mmol/L was added to a tube containing 950 µL of methanol and then incubated for 3 min at room temperature (20 °C). Then, 25 µL of the corresponding extract was added and mixed. After 30 min of incubation in the dark, the absorbance was measured at 517 nm. All experiments were performed in triplicate.

Total phenolic content: In each 2 mL tube was added 20 µL of the corresponding extract, 1580 µL of distilled water, 100 µL of Folin–Ciocalteu reagent, and 300 µL of a sodium carbonate solution at 20%. After 1 h incubation at room temperature in the dark, the absorbance was measured at 725 nm. All experiments were performed in triplicate.

FRAP, Ferric Reducing Antioxidant Power: 90 µL of distilled water, 30 µL of extract, and 900 µL of FRAP reagent at 37 °C were mixed. After 10 min of incubation at 37 °C in the dark, the absorbance was measured at 593 nm. All experiments were performed in triplicate.

### 4.4. Cell Culture

Human colon cancer cell lines SW480 (ATCC^®^ CCL-228) and SW620 (ATCC^®^ CCL-227 ™) were used. SW480 is derived from colorectal adenocarcinoma, and SW620 is derived from metastatic tissue, whose primary tumor is CRC. SW620 and SW480 cells were maintained in DMEN medium (GIBCO) supplemented with 10% fetal bovine serum and antibiotics. The cellular passages were made at 70% of cell confluence. The cells were cultured under controlled conditions at 5% CO_2_, 70% humidity, and 37 °C.

### 4.5. Cytotoxicity Studies

The cytotoxicity of tested compounds and coffee extracts was assessed using MTT and SRB assays. SW480 and SW620 cells were seeded in 96-well plates at 1.5 × 10^4^ cells/well. After 48 h, cells were treated with 100 µL of fresh medium containing different coffee extracts and compounds concentrations. After 48 h of treatments, the medium was removed and replaced with a fresh medium containing 10 µL of MTT solution (5  mg/mL), and cells were incubated for 2 h at 37 °C. Then, the MTT solution was removed, and formazan crystals were dissolved with acidified isopropyl alcohol. For SRB assays, cell monolayers were fixed with 50% (wt/vol) trichloroacetic acid and stained for 30 min, after which the excess dye was removed by repeatedly washing with 1% (vol/vol) acetic acid. Next, the protein-bound dye was dissolved in 10 mM Tris base solution for OD determination. Finally, the absorbance was read at 570 nm for MTT, and 510 nm for SRB (GloMax-Multi Detection System by Promega), and the percentage of living cells was calculated by dividing the optical density (OD) of the treated cells with the (OD) from the control cells and then multiplied by 100. For each treatment, 3 replicates were evaluated in at least 3 independent experiments.

### 4.6. Cell Migration

SW480 and SW620 cells were seeded in 24-well plates at 4 × 10^5^ cells/well without FBS supplementation for evaluation of cell migration. After 48 h, the medium was removed, and the scratch was made with a 10 µL pipette tip. After washing with PBS, cells were treated with different concentrations of the coffee extracts and compounds. The treatments were removed after 72 h, and a fresh medium was added. The images were captured at 10× with the inverted microscope and camera system (version number 4.30.01; Nikon, CA, USA) for 0–24–48–72–96 h and 120 h. The open wound area for each image was determined with Bio-EdiP software [42] and showed as a percentage of wound closure.

### 4.7. Statistical Analysis

GraphPad 6 was used to perform statistical analysis (GraphPad Software (version number 6)). Student’s *t*-test was used to assess antioxidant activity. The number of observations represents the categorical data. The variables with normal distributions were denoted by the mean and standard deviation. The variations in cell viability and open wound area were analyzed using a two-way ANOVA. *p*-value of ≤0.05 was considered statistically significant.

## 5. Conclusions

In the present study, we evaluate the cytotoxic effect of caffeic acid (CA), chlorogenic acid (CGA), green (GC), and toasted coffee (TC) extracts, and we observed that treatments decrease the viability of colorectal cancer cell lines SW480 and SW620 in a dose- and time-dependent manner. Likewise, treatments inhibit the migratory properties of cells in the wound healing assay. Furthermore, the biological impact of the phenolic compounds present in coffee that were evaluated in this study opens the possibilities of a more outstanding analysis on the mechanisms associated with these effects, which could be related to relevant therapeutic targets in the context of colorectal cancer. For these reasons, the exploration and determination of the mechanistic insights associated with the inhibition of the migration capabilities and other biological effects related to tumor development, could improve the understanding of these natural dietary compounds’ implications in treating or preventing colorectal cancer.

## Figures and Tables

**Table 1:** IC_50_ values determined by MTT and SRB methods on SW480 and SW620 cells treated with green (GC) and toasted coffee (TC) extracts, chlorogenic acid (CGA), caffeic acid (CA), and 5-fluorouracil (5-FU).

IC_50_ Value by MTT	SW480		SW620	
	24 h	48 h	24 h	48 h
Green coffee	4325 µg/mL	2555 µg/mL	9310 µg/mL	6547 µg/mL
Toasted coffee	3922 µg/mL	2226 µg/mL	3099 µg/mL	2251 µg/mL
CGA	686.6 µg/mL	598.3 µg/mL	828.6 µg/mL	841.8 µg/mL
CA	174.3 µg/mL	161.4 µg/mL	180.5 µg/mL	198.1 µg/mL
5-FU	2358 µg/mL	242.6 µg/mL	2823 µg/mL	383.3 µg/mL
**IC_50_ Values by SRB**	**SW480**		**SW620**	
	**24 h**	**48 h**	**24 h**	**48 h**
Green coffee	4676 µg/mL	2799 µg/mL	5474 µg/mL	3304 µg/mL
Toasted coffee	3656 µg/mL	1590 µg/mL	3344 µg/mL	2023 µg/mL
CGA	2844 µg/mL	1338 µg/mL	2631 µg/mL	2208 µg/mL
CA	742.8 µg/mL	460.7 µg/mL	1191 µg/mL	465.8 µg/mL
5-FU	2137 µg/mL	617.7 µg/mL	1320 µg/mL	750.6 µg/mL

## Data Availability

Data is contained within the article.
